# Author Correction: A PIP_2_ substitute mediates voltage sensor-pore coupling in KCNQ activation

**DOI:** 10.1038/s42003-022-04375-9

**Published:** 2022-12-21

**Authors:** Yongfeng Liu, Xianjin Xu, Junyuan Gao, Moawiah M. Naffaa, Hongwu Liang, Jingyi Shi, Hong Zhan Wang, Nien-Du Yang, Panpan Hou, Wenshan Zhao, Kelli McFarland White, Wenjuan Kong, Alex Dou, Amy Cui, Guohui Zhang, Ira S. Cohen, Xiaoqin Zou, Jianmin Cui

**Affiliations:** 1 Department of Biomedical Engineering, Center for the Investigation of Membrane Excitability Disorders, Cardiac Bioelectricity and Arrhythmia Center, Washington University in Saint Louis, Saint Louis, MO 63130 USA; 2 Dalton Cardiovascular Research Center, Department of Physics and Astronomy, Department of Biochemistry, Institute for Data Science & Informatics, University of Missouri, Columbia, MO 65211 USA; 3 Department of Physiology and Biophysics, and Institute for Molecular Cardiology, Stony Brook University, Stony Brook, NY 11794 USA

**Keywords:** Arrhythmias, Drug screening, Membrane biophysics

Correction to: *Communications Biology* 10.1038/s42003-020-1104-0, published online 16 July 2020.

The original version of this Article contained errors in Fig. 4, in which the labels were reversed for the “control” and “CP1” samples in panels b, g, and h. The correct version of Fig. 4 is:


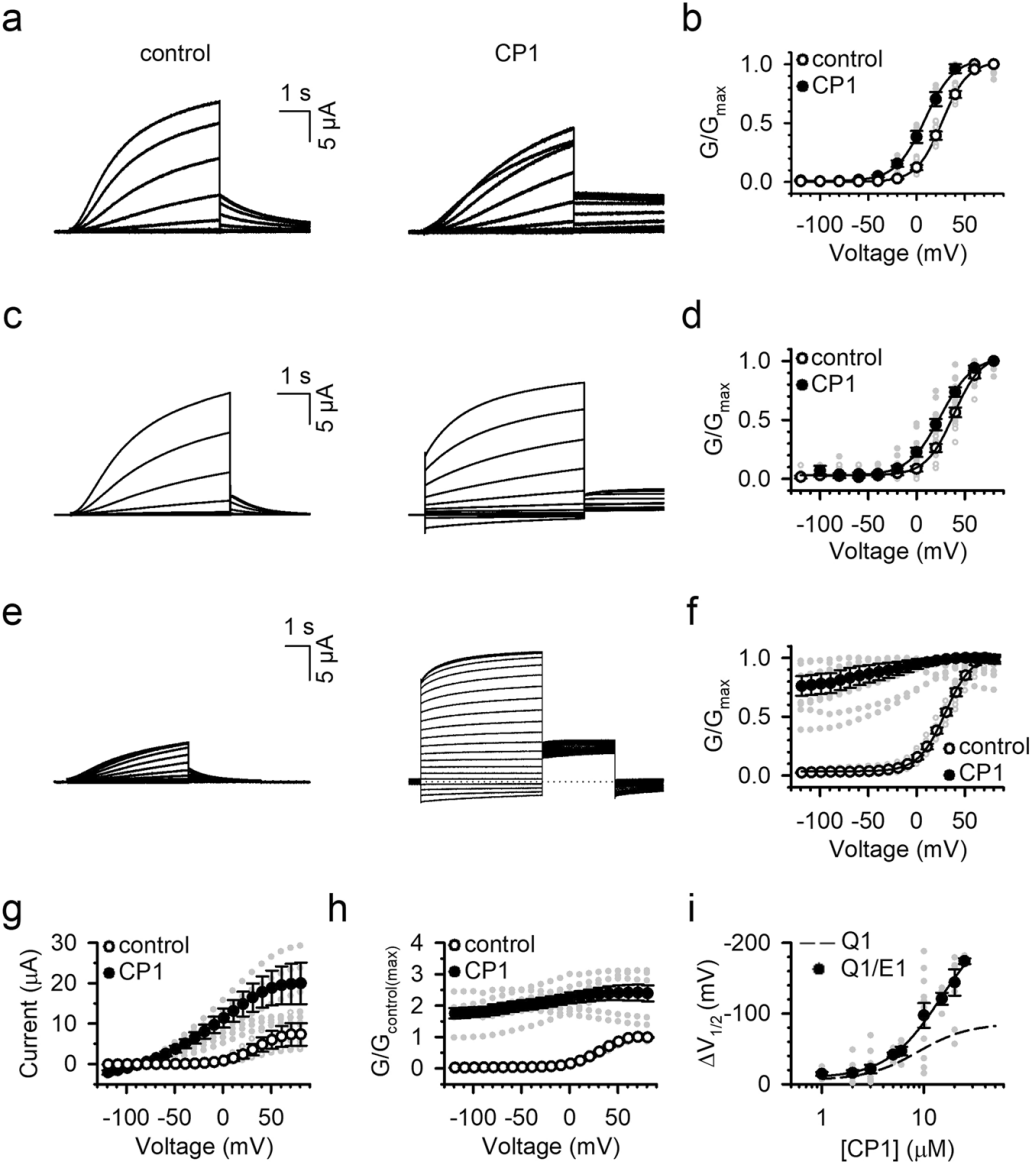


which replaces the previous incorrect version:
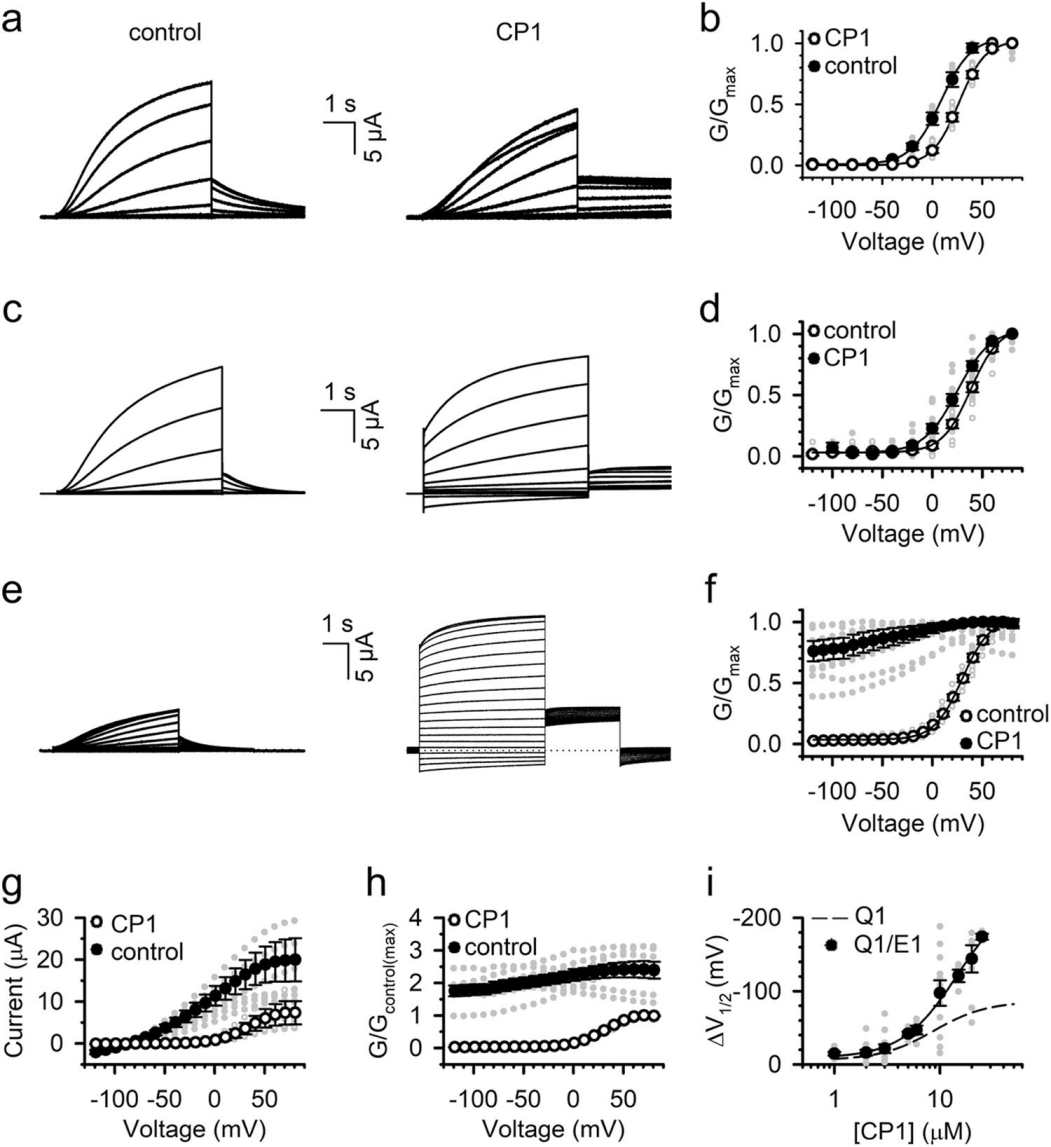


The errors have been corrected in both the PDF and HTML versions of the Article.

