# Supplementary material for: A PIP2 substitute mediates voltage sensor-pore coupling in KCNQ activation
Source: Commun Biol. 2020 Jul 16;3:385. doi: 10.1038/s42003-020-1104-0 (PMC7367283; doi:10.1038/s42003-020-1104-0)
Supplement: Supplementary file 1 — Supplementary Information [file 42003_2020_1104_MOESM1_ESM.pdf]

Supplementary Information

A PIP_2 substitute mediates voltage sensor-pore coupling in KCNQ activation

Yongfeng Liu[1, #], Xianjin Xu[2, #], Junyuan Gao[3, #], Moawiah M. Naffaa[1], Hongwu Liang[1], Jingyi Shi[1], Hong Zhan Wang[3], Nien-Du Yang[1], Panpan Hou[1], Wenshan Zhao[1], Kelli McFarland White[1], Wenjuan Kong[1], Alex Dou[1], Amy Cui[1], Guohui Zhang[1], Ira S. Cohen[3, *], Xiaoqin Zou[2, *], Jianmin Cui[1, *]

[1]Department of Biomedical Engineering, Center for the Investigation of Membrane Excitability Disorders, Cardiac Bioelectricity and Arrhythmia Center, Washington University in Saint Louis, Saint Louis, MO 63130, USA

[2]Dalton Cardiovascular Research Center, Department of Physics and Astronomy, Department of Biochemistry, Institute for Data Science & Informatics, University of Missouri, Columbia, MO 65211, USA

[3]Department of Physiology and Biophysics, and Institute for Molecular Cardiology, Stony Brook University, Stony Brook, NY 11794, USA

[#]These authors made equal contributions

[*]Corresponding author

Send correspondence for the manuscript to:

Jianmin Cui (jcui@wustl.edu),
Xiaoqin Zou (ZouX@missouri.edu), or
Ira S. Cohen (ira.cohen@stonybrook.edu)

Supplementary table 1. Primer sequence information in this study.

Mutations	Primer sequences (5’-3’)
R243A	pB: ctccctggGCAtcgacgtgtagcatc<br>pC: cgtcgaTGCccagggaggcacctg
W248R	pB: cctccGggtgcctccctggc<br>pC: gaggcaccCggaggctcctgggc
R249A	pB: gagcGCccaCgtgcctccctggcg<br>pC: gaggcacGtggGCgctcctgggctccgt
S253A	pB: gaccacAGCgcccaggagcctcc<br>pC: ctgggcGCTgtggtcttcatccacc
H258A	pB: cctggcgCGCgatgaagaccacggag<br>pC: catcGCGcgccaggagctgataac
R259A	pB: ctcctgTGCAtggatgaagaccacgg<br>pC: catccaTGCAcaggagctgataaccac
K354A	pB: ctgcacGGCcagggcaaaccccg<br>pC: ccctgGCCgtgcagcagaagcag
K358A	pB: gtgcagcaggcgcagaggcagaagcac<br>pC: gtgcttctgcctctgcgcctgctgcac
Q359A	pB: gtgcagcagaaggcgaggcagaagcac<br>pC: gtgcttctgcctcgccttctgctgcac
R243A/R249A	pB: ctccctggGCAtcgacgtgtagcatc<br>pC: cgtcgaTGCccagggaggcacGtg
R243A/R259A	Same as R243A and R259A
R249A/S253A	pB: gaccacAGCgcccaggagcGCcc<br>pC: ctgggcGCTgtggtcttcatccacc
R249A/K354A	Same as R249A and K354A
K354A/K358A	pB: ctgcacGGCcagggcaaaccccg<br>pC: ccctgGCCgtgcagcagGCgcag
R249A/S253A/K354A/K358A	Same as R249A/S253A and K354A/K358A

Supplementary Figure 1

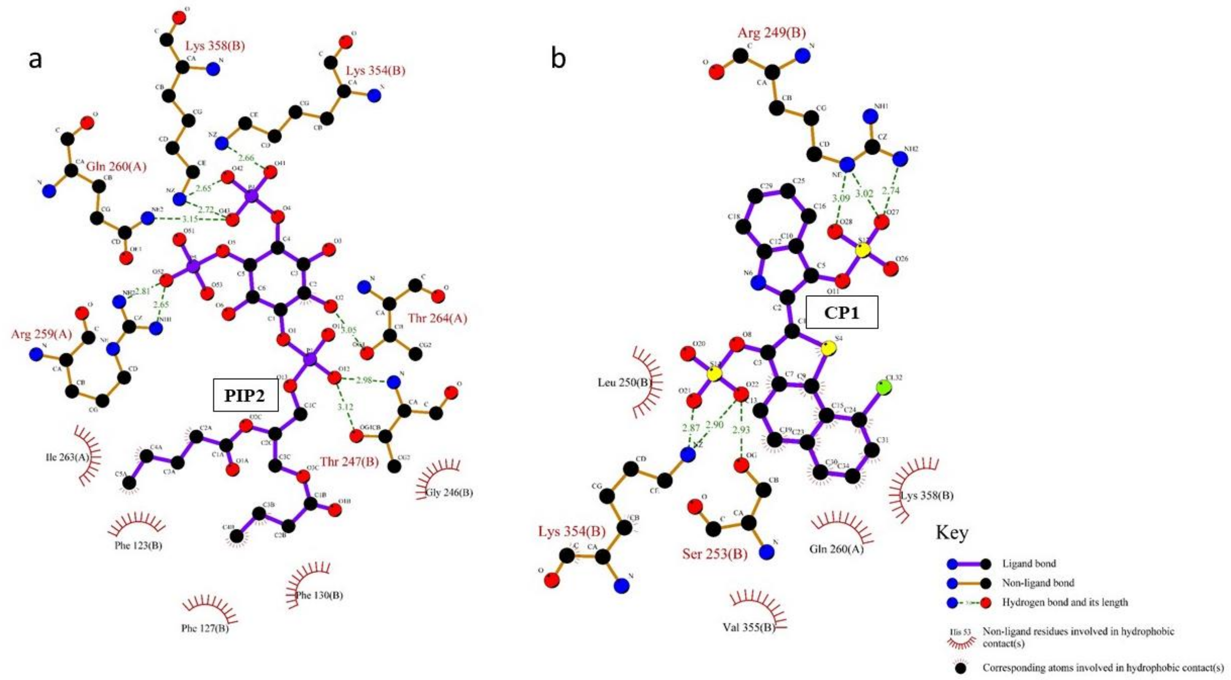

Supplementary fig. 1 Interaction details of PIP_2 and CP1 with human KCNQ1 channel. Interaction details of PIP_2 (a) and CP1 (b) with human KCNQ1, respectively. The interacting residues were determined using the LIGPLOT program[1] based on the predicted complex structures (also shown in Fig. 1b). The letter in the parenthesis after each residue number shows the corresponding chain id. Experimentally confirmed critical residues for the ligand binding are represented in Fig. 1b.

Supplementary Figure 2

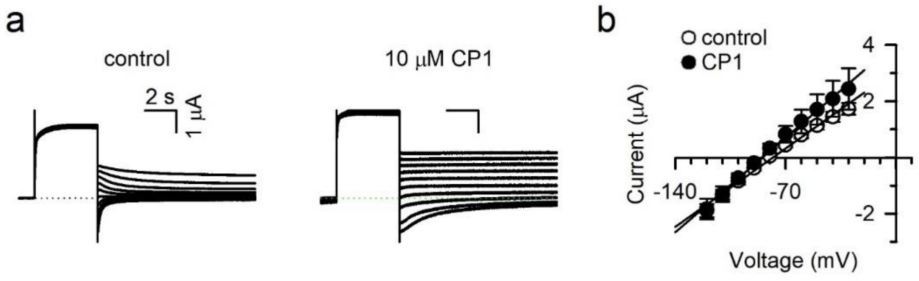

Supplementary fig. 2 CP1 did not change the reversal potential of KCNQ1 channels. (**a**) Representative traces of KCNQ1 currents elicited in the absence and presence of 10 µM CP1. From a holding potential of -80 mV, the KCNQ1 currents were elicited at +40 mV. The deactivated tail currents were elicited by 10 s test pulses between -120 mV to -30 mV in 10 mV step. (b) Current-voltage relations of KCNQ1 channels. The reversal potential for control and 10 µM CP1 is -78.0 ± 2.0 mV and -83.4 ± 2.2 mV, respectively.

Supplementary Figure 3

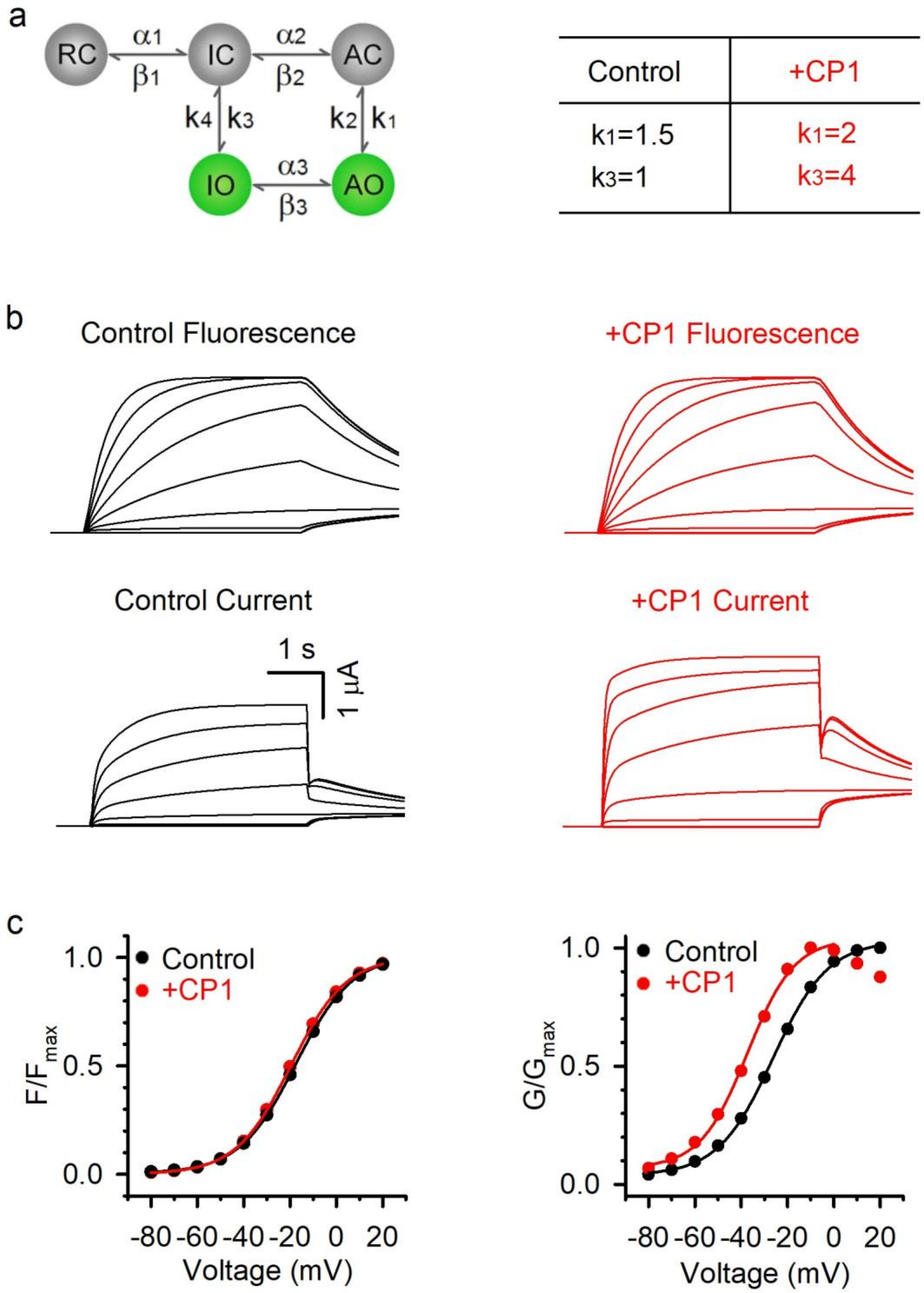

Supplementary fig. 3 Model simulations to illustrate that an alteration of the coupling between the voltage sensor domain and the pore may cause a larger shift in G-V than in F-V. (**a**) The five-state Markov model that was proposed by previous studies to describe the gating processes of the KCNQ1 channel involving two open states[2,3]. α and β are voltage-dependent

transition rates among the resting (R), intermediate (I) and activated (A) states of the voltage sensor domain (VSD) movements. k1-4 are closed-open transition rates when the VSD is at either the intermediate (I) or activated (A) state (see Methods), which represent the VSD-pore coupling. Increasing k1 and k3 (table at right) may qualitatively illustrate the CP1 effects on the G-V and F-V shifts of the KCNQ1 channel. β3 is also changed to balance the model (see Methods). (b) Model simulations of KCNQ1 VSD activation (fluorescence) and pore opening (current) before (black) and after (red) the changes in k1 and k3 (a, right) that mimic the addition of CP1. (c) F–V and G–V relationships of simulated KCNQ1 before (black) and after (red) the changes in k1 and k3 (a, right) that mimic the addition of CP1. Data points were fitted with a Boltzmann function (for F-V, $V_{1/2}$ = = -17.8 mV and -19.6 mV, slope factor = 12.0 mV and 11.8 mV in control and CP1, respectively; for G-V, $V_{1/2}$ = -27.6 mV and -40.3 mV, slope factor = 11.3 mV and 9.5 mV in control and CP1, respectively). The F–V shows a left-shift of 1.8 mV, while the G–V shows a left-shift of 12.7 mV as the result of the changes in k1 and k3.

Supplementary Figure 4

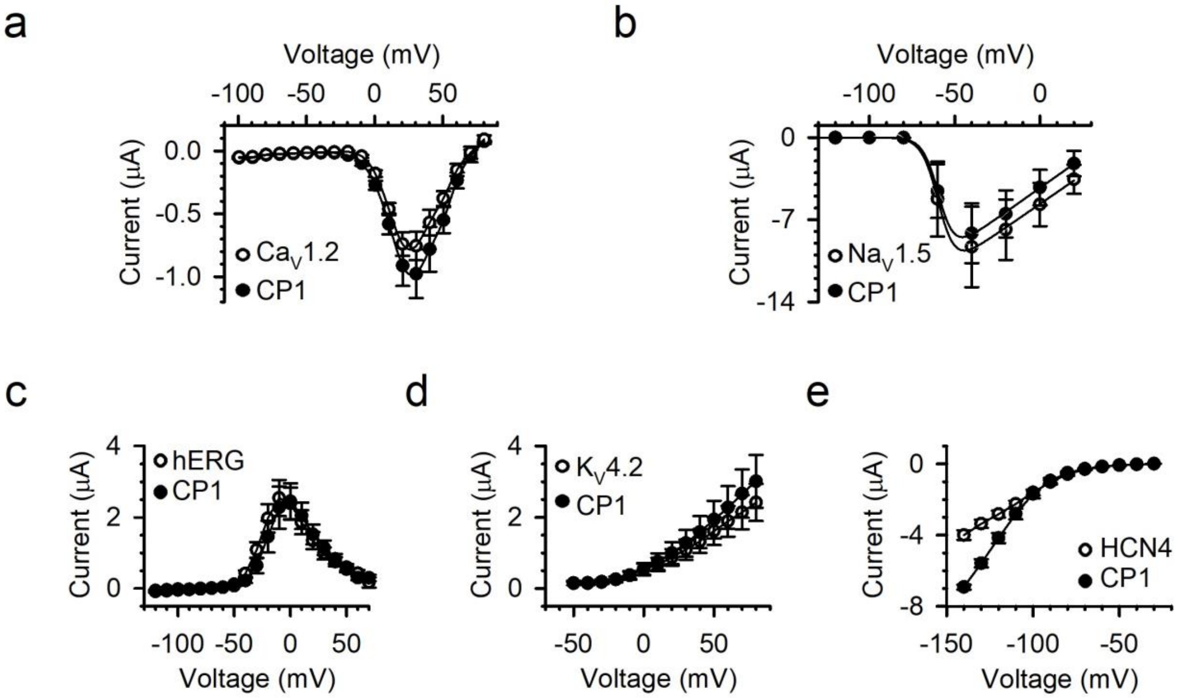

Supplementary Fig. 4 Current-voltage relations of indicated channel in the absence or presence of 10 μM CP1. (**a**) $Ca_V1.2$; (**b**) $Na_V1.5$; (**c**) hERG; (**d**) $K_V4.2$; and (**e**) HCN4.

Supplementary Figure 5.

	S4 … S5	S6
KCNQ1	RMLHVDRQGGTWRLLGSVVFIHRQELITT	GSGFALKVQQKQRQKHFNRQI
KCNQ2	RMIRMDRRGGTWKLLGSVVYAHSKELVTA	GSGFALKVQEQHRQKHFEKRR
KCNQ3	RMLRMDRRGGTWKLLGSAICAHSKELITA	GSGLALKVQEQHRQKHFEKRR
Cav1.2(I)	RLVSGVPSLQVVLNSIIKAMVPLLHIALL	LNLVLGVLSGEFSKEREKAKA
Cav1.2(II)	KITRYWNSLSNLVASLLNSVRSIASLLLL	LNVFLAIAVDNLADAESLTSA
Cav1.2(III)	RAINRAKGLKHVVQCVFVAIRTIGNIVIV	MNIFVGFVIVTFQEQGEQEYK
Cav1.2(IV)	KLLSRGEGIRTLLWTFIKSFQALPYVALL	INLFVAVIMDNFDYLTRDWSI
Nav1.5(I)	KTISVISGLKTIVGALIQSVKKLADVMVL	VNLILAVVAMAYEEQNQATIA
Nav1.5(II)	KLAKSWPTLNTLIKIIGNSVGALGNLTLV	LNLFLALLLSSFSADNLTAPD
Nav1.5(III)	RALSRFEGMRVVVNALVGAIPSIMNVLLV	LNLFIGVIIDNFNQQKKKLGG
Nav1.5(IV)	RLIRGAKGIRTLLFALMMSLPALFNIGLL	VNMYIAIILENFSVATEESTE
Kv4.2	KFSRHSQGLRILGYTLKSCASELGFLLFS	VSNFSRIYHQNQRADKRRAQK
HCN4	RYIHQWEEIFHMTYDLASAVVRIVNLIGM	AMFIGHATALIQSLDSSRRQY
hERG	RVARKLDRYSEYG---------AAVLFLL	ASIFGNVSAIIQRLYSGTARY

Supplementary Fig. 5 Sequence alignment of S4-S5L and S6C of human voltage-gated ion channels. The putative CP1 interaction residues are highlighted blue in KCNQ sequences. The channels outside of the KCNQ family exhibit low sequence homology.

Reference

1 Wallace, A. C., Laskowski, R. A. & Thornton, J. M. LIGPLOT: a program to generate schematic diagrams of protein-ligand interactions. *Protein Eng* **8**, 127-134, doi:10.1093/protein/8.2.127 (1995).

2 Hou, P. *et al.* Inactivation of KCNQ1 potassium channels reveals dynamic coupling between voltage sensing and pore opening. *Nature communications* **8**, 1730, doi:10.1038/s41467-017-01911-8 (2017).

3 Zaydman, M. A. *et al.* Domain-domain interactions determine the gating, permeation, pharmacology, and subunit modulation of the IKs ion channel. *eLife* **3**, e03606, doi:10.7554/eLife.03606 (2014).
