# Supplementary material for: A PIP2 substitute mediates voltage sensor-pore coupling in KCNQ activation
Source: Commun Biol. 2020 Jul 16;3:385. doi: 10.1038/s42003-020-1104-0 (PMC7367283; doi:10.1038/s42003-020-1104-0)
Supplement: Supplementary file 5 — Reporting Summary [file 42003_2020_1104_MOESM5_ESM.pdf]

nature research

Corresponding author(s): Jianmin Cui, Xiaoqin Zou, Ira S. Cohen

Last updated by author(s): Jun 2, 2020

Reporting Summary

Nature Research wishes to improve the reproducibility of the work that we publish. This form provides structure for consistency and transparency in reporting. For further information on Nature Research policies, see Authors & Referees and the Editorial Policy Checklist.

Statistics

For all statistical analyses, confirm that the following items are present in the figure legend, table legend, main text, or Methods section.

n/a	Confirmed	
☐	☒	The exact sample size (n) for each experimental group/condition, given as a discrete number and unit of measurement
☐	☒	A statement on whether measurements were taken from distinct samples or whether the same sample was measured repeatedly
☐	☒	The statistical test(s) used AND whether they are one- or two-sided<br>*Only common tests should be described solely by name; describe more complex techniques in the Methods section.*
☐	☒	A description of all covariates tested
☐	☒	A description of any assumptions or corrections, such as tests of normality and adjustment for multiple comparisons
☐	☒	A full description of the statistical parameters including central tendency (e.g. means) or other basic estimates (e.g. regression coefficient) AND variation (e.g. standard deviation) or associated estimates of uncertainty (e.g. confidence intervals)
☐	☒	For null hypothesis testing, the test statistic (e.g. F, t, r) with confidence intervals, effect sizes, degrees of freedom and P value noted<br>*Give P values as exact values whenever suitable.*
☒	☐	For Bayesian analysis, information on the choice of priors and Markov chain Monte Carlo settings
☒	☐	For hierarchical and complex designs, identification of the appropriate level for tests and full reporting of outcomes
☒	☐	Estimates of effect sizes (e.g. Cohen's d, Pearson's r), indicating how they were calculated

Our web collection on statistics for biologists contains articles on many of the points above.

Software and code

Policy information about availability of computer code

Data collection	Data for two-electrode voltage clamp, voltage clamp fluorometry and inside-out patch clamp were all collected with the Patchmaster software (HEKA, Germany). Data for cut-open voltage clamp were collected with the Clampex software (v10; Molecular Devices). Data for whole-cell patch clamp were collected with pClamp software.
Data analysis	Electrophysiology data were processed with IGOR 6.03 (Wavemetrics, Lake Oswego, OR), Clampfit 10.7 (Molecular Devices, Sunnyvale, CA), SigmaPlot 14.0 (SPSS, Inc., San Jose, CA).

For manuscripts utilizing custom algorithms or software that are central to the research but not yet described in published literature, software must be made available to editors/reviewers. We strongly encourage code deposition in a community repository (e.g. GitHub). See the Nature Research guidelines for submitting code & software for further information.

Data

Policy information about availability of data

All manuscripts must include a data availability statement. This statement should provide the following information, where applicable:

- Accession codes, unique identifiers, or web links for publicly available datasets
- A list of figures that have associated raw data
- A description of any restrictions on data availability

All the experimental data generated or analyzed during this study are present in the paper. Additional data and research materials related to this paper are available from the corresponding author on reasonable request.

Field-specific reporting

Please select the one below that is the best fit for your research. If you are not sure, read the appropriate sections before making your selection.

☒ Life sciences ☐ Behavioural & social sciences ☐ Ecological, evolutionary & environmental sciences

For a reference copy of the document with all sections, see nature.com/documents/nr-reporting-summary-flat.pdf

Life sciences study design

All studies must disclose on these points even when the disclosure is negative.

Sample size	Each electrophysiology experiment was performed on at least 3 individual cells. The number of recordings of each experiment was based on personal experience and data variability.
Data exclusions	No data was excluded from the analysis.
Replication	Electrophysiology experiments were each performed on at least two separate batches of oocytes or myocytes to confirm reproducibility.
Randomization	For this study, self controlled case series methods were used to the data collection and analysis. The control and covariates were defined with before and after CP1 treatment.
Blinding	The investigators were not blinded to allocation during experiments and outcome assessment.

Reporting for specific materials, systems and methods

We require information from authors about some types of materials, experimental systems and methods used in many studies. Here, indicate whether each material, system or method listed is relevant to your study. If you are not sure if a list item applies to your research, read the appropriate section before selecting a response.

Materials & experimental systems

n/a	Involved in the study	
☒	☐	Antibodies
☒	☐	Eukaryotic cell lines
☒	☐	Palaeontology
☐	☒	Animals and other organisms
☒	☐	Human research participants
☒	☐	Clinical data

Methods

n/a	Involved in the study	
☒	☐	ChIP-seq
☒	☐	Flow cytometry
☒	☐	MRI-based neuroimaging

Animals and other organisms

Policy information about studies involving animals; ARRIVE guidelines recommended for reporting animal research

Laboratory animals	Xenopus laevis, female; Guinea pig, male or female, weighting 300-500 g
Wild animals	This study did not involve wild animals.
Field-collected samples	This study did not involve filed-collected samples.
Ethics oversight	The use of Xenopus laevis to obtain oocytes was approved by the Washington University Animal Studies Committee (protocol #20190030). The use of Guinea Pig to obtain single ventricular myocytes was approved by the Stony Brook University Institutional Animal Care and Use Committee.

Note that full information on the approval of the study protocol must also be provided in the manuscript.
